# Unsupervised clustering identifies sub-phenotypes and reveals novel outcome predictors in patients with dialysis-requiring sepsis-associated acute kidney injury

**DOI:** 10.1080/07853890.2023.2197290

**Published:** 2023-04-12

**Authors:** Chun-Fu Lai, Jung-Hua Liu, Li-Jung Tseng, Chun-Hao Tsao, Nai-Kuan Chou, Shuei-Liong Lin, Yung-Ming Chen, Vin-Cent Wu

**Affiliations:** a Renal Division, Department of Internal Medicine, National Taiwan University Hospital, Taipei City, Taiwan; b Department of Communication, National Chung Cheng University, Minhsiung, Taiwan; c Department of Surgery, National Taiwan University Hospital, Taipei City, Taiwan; d Graduate Institute of Physiology, National Taiwan University College of Medicine, Taipei City, Taiwan; e National Taiwan University Hospital Bei-Hu Branch, Taipei City, Taiwan

**Keywords:** Acute kidney injury, cluster analysis, competing risk, recovery of function, renal replacement therapy, Sepsis-3, sequential organ failure assessment

## Abstract

**Introduction:**

Heterogeneity exists in sepsis-associated acute kidney injury (SA-AKI). This study aimed to perform unsupervised consensus clustering in critically ill patients with dialysis-requiring SA-AKI.

**Patients and Methods:**

This prospective observational cohort study included all septic patients, defined by the Sepsis-3 criteria, with dialysis-requiring SA-AKI in surgical intensive care units in Taiwan between 2009 and 2018. We employed unsupervised consensus clustering based on 23 clinical variables upon initializing renal replacement therapy. Multivariate-adjusted Cox regression models and Fine–Gray sub-distribution hazard models were built to test associations between cluster memberships with mortality and being free of dialysis at 90 days after hospital discharge, respectively.

**Results:**

Consensus clustering among 999 enrolled patients identified three sub-phenotypes characterized with distinct clinical manifestations upon renal replacement therapy initiation (*n* = 352, 396 and 251 in cluster 1, 2 and 3, respectively). They were followed for a median of 48 (interquartile range 9.5–128.5) days. Phenotypic cluster 1, featured by younger age, lower Charlson Comorbidity Index, higher baseline estimated glomerular filtration rate but with higher severity of acute illness was associated with an increased risk of death (adjusted hazard ratio of 3.05 [95% CI, 2.35–3.97]) and less probability to become free of dialysis (adjusted sub-distribution hazard ratio of 0.55 [95% CI, 0.38–0.8]) than cluster 3. By examining distinct features of the sub-phenotypes, we discovered that pre-dialysis hyperlactatemia ≥3.3 mmol/L was an independent outcome predictor. A clinical model developed to determine high-risk sub-phenotype 1 in this cohort (C-static 0.99) can identify a sub-phenotype with high in-hospital mortality risk (adjusted hazard ratio of 1.48 [95% CI, 1.25–1.74]) in another independent multi-centre SA-AKI cohort.

**Conclusions:**

Our data-driven approach suggests sub-phenotypes with clinical relevance in dialysis-requiring SA-AKI and serves an outcome predictor. This strategy represents further development toward precision medicine in the definition of high-risk sub-phenotype in patients with SA-AKI.

**Key messages**
1. Unsupervised consensus clustering can identify sub-phenotypes of patients with SA-AKI and provide a risk prediction.
2. Examining the features of patient heterogeneity contributes to the discovery of serum lactate levels ≥ 3.3 mmol/L upon initializing RRT as an independent outcome predictor.
3. This data-driven approach can be useful for prognostication and lead to a better understanding of therapeutic strategies in heterogeneous clinical syndromes.

## Introduction

Acute kidney injury (AKI) is common in sepsis [1–3]. Patients with sepsis-associated (SA) AKI have a greater burden of illness and abnormalities in haemodynamics and laboratory parameters than those with non-septic AKI [4]. We and others have shown that the presence of SA-AKI is strongly associated with increased short- and long-term mortality [4,5]. Furthermore, compared with patients who did not require dialysis, those who underwent dialysis during SA-AKI had a much higher risk of mortality[2] and lower possibility of kidney recovery [6]. Recent studies reported that 90-day mortality in patients with dialysis-requiring SA-AKI ranged from 45.2% to 81.2% [7–10]. Therefore, this population represents the highest risk of adverse clinical outcomes.

Both sepsis and AKI are highly heterogenous syndromes [11,12]. It is not surprising that several reports addressed the presence of heterogeneity in aetiology, disease trajectory, prognosis and outcomes among patients with SA-AKI [13,14]. Furthermore, diverse pathophysiological mechanisms of SA-AKI are not well-understood yet [15,16]. These features make clinical managements of SA-AKI not optimal or standardized [3,17]. Unsupervised clustering is an agnostic class discovery largely used in basic experiments and genetic studies [18]. This data-driven approach has been successfully applied in clinical studies to identify distinct sub-phenotypes in diseases with high heterogeneity, such as heart failure [19], hypertension [20], diabetes [21], sepsis [22] and chronic kidney disease [23]. Literature suggests unsupervised clustering can identify sub-phenotypes for outcome predictions in patients with SA-AKI [24–26]. However, none of these clinical studies further deciphered previously unrecognized markers or mechanisms hidden behind the sub-phenotypes of SA-AKI, while basic research always did so [27,28].

We hypothesized that agnostically clustering patients with dialysis-requiring SA-AKI can identify distinct subpopulations with different clinical relevance. This study was designed to examine phenotype heterogeneity in critically ill patients with dialysis-requiring SA-AKI using consensus clustering of multidimensional clinical data. Understanding potential predictors of clinical outcomes may facilitate appropriate intervention and contribute to early shared decision-making for caring patients with SA-AKI.

## Patients and Methods

### Study cohort

The main study cohort, National Taiwan University Hospital Study Group on Acute Renal Failure (NSARF) database contains prospectively collected data from patients with AKI during surgical intensive care unit (ICU) admission [29–32]. Enrolled patients were admitted to surgical services for perioperative care or other unstable medical conditions due to sepsis. The study was complied with the Helsinki Declaration and approved by the Institutional Review Board of National Taiwan University Hospital (201407076RINA). Written informed consent was waived because there was no breach of privacy or interference with patient care.

In this observational cohort study, we investigated patients with dialysis-requiring SA-AKI, defined by the simultaneous presence of both sepsis and AKI [33]. We first screened patients aged 18 years and above in the database who encountered an episode of dialysis-requiring AKI and had suspected concurrent infection between January 1, 2009 and December 31, 2018. Dialysis-requiring AKI was defined as the need to receive renal replacement therapy (RRT) during the index AKI according to the Kidney Disease: Improving Global Outcomes AKI criteria [34]. Patients who had pre-existing end-stage kidney disease, were kidney transplant recipients or had ever received any RRT were excluded from the study. Infection was suspected as the combination of usage of systemic antibiotics and positive microbiology culture from body fluids. Sepsis was defined according to the third international consensus definition for sepsis and septic shock (Sepsis-3) [35]. To be classified as having sepsis, patients with a suspected or confirmed infection should have met at least two quick Sequential Organ Failure Assessment (SOFA) criteria and have an acute increase in total SOFA score by ≥2 within the 24-h period before RRT was started. The baseline SOFA component was considered zero unless the patient has pre-existing organ dysfunction before the onset of infection [35]. Patients with chronic hepatic dysfunction and chronic respiratory impairment were assigned a baseline SOFA score of 4 and 2, respectively [36].

### Clinical assessments and dialysis initiation

All demographic and clinical data were prospectively collected on a standardized form. Baseline serum creatinine was defined as the lowest value measured within 180 days before the index admission, or the lowest value before dialysis during the index admission if the patient had no serum creatinine measurement within the previous 180-day period [30,37]. Baseline estimated glomerular filtration rate (eGFR) was calculated by the four-variable Modification of Diet in Renal Disease equation [38]. The worst physiological and laboratory values during the 24-h period upon initializing RRT were collected. Missing data was imputed using Multivariate Imputation by Chained Equation. The doses of inotropic therapies were expressed as inotropic equivalent (IE) (μg/kg/min) [39]. The details of the collected parameters are shown in Table 1 and Supplemental Methods. RRT was initiated according to the pre-determined indications detailed in the Supplemental Methods.

**Table 1.** Patients characteristics.

(A) Demographic characteristics at baseline						
Variable	Main study cohort (NSARF)					**External cohort** **(NEP-AKI-D)** **(*n* = 898)**
	**Overall** **(*n* = 999)**	**Cluster 1** **(*n* = 352)**	**Cluster 2** **(*n* = 396)**	**Cluster 3** **(*n* = 251)**	*P* Value^a^	
Age, years	63.87 ± 16.51	57.82 ± 15.68	62.87 ± 16.29	73.95 ± 12.98	<0.001	66.94 ± 15.68
Male sex	714 (71.47%)	252 (71.59%)	292 (73.74%)	170 (67.73%)	0.26	576 (64.14%)
Current smoker or ex-smoker	182 (18.22%)	71 (20.17%)	71 (17.93%)	40 (15.94%)	0.41	155 (17.26%)
Diabetes mellitus	405 (40.54%)	109 (30.97%)	141 (35.61%)	155 (61.75%)	<0.001	455 (50.67%)
Hypertension	590 (59.06%)	173 (49.15%)	219 (55.3%)	198 (78.88%)	<0.001	523 (58.24%)
Coronary artery disease	333 (33.33%)	97 (27.56%)	138 (34.85%)	98 (39.04%)	0.01	207 (23.05%)
Stroke	79 (7.91%)	16 (4.55%)	32 (8.08%)	31 (12.35%)	0.002	114 (12.69%)
Peripheral arterial occlusive disease	46 (4.6%)	6 (1.7%)	19 (4.8%)	21 (8.37%)	0.001	65 (7.24%)
Advanced heart failure, NYHA Fc III or IV	222 (22.22%)	85 (24.15%)	79 (19.95%)	58 (23.11%)	0.36	152 (16.93%)
Chronic obstructive pulmonary disease	52 (5.21%)	12 (3.41%)	23 (5.81%)	17 (6.77%)	0.15	64 (7.13%)
Liver cirrhosis	114 (11.41%)	42 (11.93%)	46 (11.62%)	26 (10.36%)	0.83	132 (14.7%)
Cancer	225 (22.52%)	65 (18.47%)	86 (21.72%)	74 (29.48%)	0.005	247 (27.51%)
Charlson Comorbidity Index	5.3 ± 2.28	4.39 ± 1.98	5.24 ± 2.26	6.65 ± 2.02	<0.001	6.76 ± 3.13
Baseline eGFR, mL/min/1.73m^2^	58.79 ± 34.92	74.6 ± 32.74	61.99 ± 30.39	31.56 ± 28.11	<0.001	64.42 ± 40.17
Index admission year before 2014	393 (39.34%)	150 (42.61%)	163 (41.16%)	80 (31.87%)	0.02	0
Surgery	498 (49.85%)	185 (52.56%)	221 (55.81%)	92 (36.65%)	<0.001	196 (21.83%)
Abdominal surgery	102 (10.21%)	27 (7.67%)	45 (11.36%)	30 (11.95%)	0.14	69 (7.68%)
Cardiovascular surgery	252 (25.23%)	106 (30.11%)	116 (29.29%)	30 (11.95%)	<0.001	55 (6.12%)
Chest surgery	24 (2.4%)	13 (3.69%)	9 (2.27%)	2 (0.8%)	0.07	7 (0.78%)
Neurology surgery	13 (1.3%)	2 (0.57%)	3 (0.76%)	8 (3.17%)	0.009	12 (1.34%)
Trauma surgery	6 (0.6%)	2 (0.57%)	3 (0.76%)	1 (0.4%)	0.84	6 (0.67%)
Shock	614 (61.46%)	265 (75.28%)	251 (63.38%)	98 (37.85%)	<0.001	600 (66.82%)
Contrast exposure	103 (10.31%)	38 (10.8%)	37 (9.34%)	28 (11.16%)	0.71	54 (6.01%)
Nephrotoxic drug	9 (0.9%)	2 (0.57%)	4 (1.01%)	3 (1.2%)	0.69	45 (5.01%)
Total parenteral nutrition	171 (17.12%)	57 (16.19%)	73 (18.43%)	41 (16.33%)	0.67	n.a.
Mechanical ventilation	795 (79.58%)	328 (93.18%)	310 (78.28%)	157 (62.55%)	<0.001	779 (86.75%)
Cardiopulmonary resuscitation	289 (28.93%)	134 (38.07%)	102 (25.76%)	53 (21.12%)	<0.001	132 (14.7%)
ECMO	409 (40.94%)	219 (62.22%)	148 (37.37%)	42 (16.73%)	<0.001	n.a.
Source of sepsis						
Respiratory tract	679 (67.97%)	235 (66.76%)	271 (68.43%)	173 (68.92%)	0.83	555 (61.8%)
Intra-abdominal	38 (3.8%)	8 (2.27%)	19 (4.8%)	11 (4.38%)	0.17	128 (14.25%)
Skin or soft-tissue	104 (10.41%)	32 (9.09%)	52 (13.13%)	20 (7.97%)	0.07	34 (3.79%)
Genitourinary tract	112 (11.21%)	42 (11.93%)	45 (11.36%)	25 (9.96%)	0.75	349 (38.86%)
Device or catheter associated	238 (23.82%)	91 (25.85%)	97 (24.49%)	50 (19.92%)	0.22	n.a.
Blood stream	171 (17.12%)	66 (18.75%)	68 (17.17%)	37 (14.74%)	0.44	285 (31.74%)
Others/unknown	276 (27.63%)	96 (27.27%)	110 (27.78%)	70 (27.89%)	0.98	80 (8.91%)
First RRT within 2 days of hospital admission	341 (34.13%)	152 (43.18%)	123 (31.06%)	66 (26.29%)	<0.001	318 (35.41%)
Indication for RRT initiation						
Azotaemia with symptoms	486 (48.65%)	108 (30.68%)	189 (47.73%)	189 (75.3%)	<0.001	483 (53.79%)
Fluid overload	511 (51.15%)	184 (52.27%)	214 (54.04%)	113 (45.02%)	0.07	552 (61.47%)
Electrolyte imbalance	135 (13.51%)	59 (16.76%)	46 (11.62%)	30 (11.95%)	0.09	318 (35.41%)
Metabolic acidosis	256 (25.63%)	126 (35.8%)	75 (18.94%)	55 (21.91%)	<0.001	454 (50.56%)
Oliguria	662 (66.27%)	257 (73.01%)	285 (71.97%)	120 (47.81%)	<0.001	567 (63.14%)
Others	71 (7.11%)	37 (10.51%)	28 (7.07%)	6 (2.39%)	0.001	90 (10.02%)
Modality at the first RRT					<0.001	
CRRT	598 (59.86%)	291 (82.67%)	217 (54.8%)	90 (35.86%)		337 (37.53%)
Sustained low-efficiency dialysis	212 (21.22%)	46 (13.07%)	89 (22.47%)	77 (30.68%)		45 (5.01%)
Intermittent haemodialysis	189 (18.92%)	15 (4.26%)	90 (22.73%)	84 (33.47%)		404 (44.99%)
Mixed	n.a.					109 (12.14%)
(B) Baseline clinical data upon initializing RRT						
Variable	Main study cohort (NSARF)					**External cohort** **(NEP-AKI-D)** **(*n* = 898)**
	**Overall** **(*n* = 999)**	**Cluster 1** **(*n* = 352)**	**Cluster 2** **(*n* = 396)**	**Cluster 3** **(*n* = 251)**	*P* Value^a^	
log_e_ (urine volume, mL/day)	5.19 ± 1.94	4.98 ± 1.99	5.06 ± 1.95	5.68 ± 1.77	<0.001	5.13 ± 2.06
Glasgow Coma Scale	8.33 ± 4.02	4.62 ± 2.37	10.57 ± 3.12	10.01 ± 3.33	<0.001	7.88 ± 3.98
Body weight	69.08 ± 16.88	73.25 ± 18.21	67.74 ± 16.27	65.36 ± 14.56	<0.001	67.6 ± 14.83
Body temperature, degree Celsius	36.53 ± 1.15	36.57 ± 1.35	36.59 ± 1.06	36.4 ± 0.93	0.09	36.52 ± 1.23
Heart rate, beats per minute	100.95 ± 20.76	106.89 ± 21.18	101.87 ± 18.42	91.18 ± 20.18	<0.001	102.16 ± 23.21
Mean arterial pressure, mm Hg	78.27 ± 16.15	76.11 ± 17.09	78.2 ± 14.45	81.42 ± 16.86	<0.001	78.34 ± 18.13
log_e_(ratio of PaO2 to fraction of inspired oxygen)	5.35 ± 0.7	5.06 ± 0.75	5.57 ± 0.6	5.39 ± 0.65	<0.001	5.36 ± 0.7
Blood urea nitrogen, mg/dL	75.93 ± 45.08	54.49 ± 36.81	71.21 ± 39.48	113.45 ± 40.63	<0.001	87.1 ± 50.21
Serum creatinine, mg/dL	3.89 ± 2.4	2.75 ± 1.6	3.52 ± 1.98	6.08 ± 2.5	<0.001	4.69 ± 2.64
Sodium, mmol/L	139.58 ± 8.33	141.84 ± 8.94	138.85 ± 7.65	137.56 ± 7.76	<0.001	139.45 ± 8.55
Potassium, mmol/L	4.32 ± 0.9	4.26 ± 0.94	4.28 ± 0.78	4.48 ± 0.99	0.005	4.56 ± 1.15
White blood cells, 10^3^cells/μL	13.89 ± 8.13	14.25 ± 7.56	13.59 ± 7.67	13.85 ± 9.51	0.54	15.35 ± 11.92
Haemoglobin, g/dL	10.16 ± 2.21	11.01 ± 2.53	10.15 ± 1.93	8.99 ± 1.5	<0.001	9.61 ± 2.22
Platelets, 10^3^cells/μL	128.51 ± 88.47	117.35 ± 84.3	112.72 ± 79.11	169.09 ± 95.69	<0.001	134.11 ± 102.85
Total bilirubin, mg/dL	4.06 ± 6.97	3.97 ± 5.56	5.42 ± 8.46	2.02 ± 5.5	<0.001	3.38 ± 6.01
Bicarbonate, mmol/L	18.71 ± 4.9	18.56 ± 5.07	18.95 ± 4.69	18.52 ± 5	0.43	17.98 ± 5.66
Lactate, mmol/L	5.19 ± 5.53	7.62 ± 6.29	4.65 ± 5.07	2.61 ± 3.27	<0.001	5.74 ± 6.67
Inotropic equivalent, μg/kg/min (median [IQR])	7.7 (0–19.25)	14.1 (6.22–28.47)	7.36 (0–18.1)	0 (0–7.1)	<0.001	5.06 (0–21.58)
APACH II score (median [IQR])	21 (17–25)	25 (22–28.25)	18 (14–20)	22 (18–25)	<0.001	24 (20–29)
SOFA score (median [IQR])	13 (10–15)	16 (14–17)	13 (10–15)	10 (8–13)	<0.001	13 (10–16)

NSARF: National Taiwan University Hospital Study Group on Acute Renal Failure database; NEP-AKI-D: nationwide epidemiology and prognosis of dialysis-requiring acute kidney injury study; NYHA Fc: New York Heart Association functional class; eGFR: estimated glomerular filtration rate; n.a.: not available; ECMO: extracorporeal membrane oxygenation; RRT: renal replacement therapy; CRRT: continuous renal replacement therapy; RRT: renal replacement therapy; IQR: interquartile range; APACH II: Acute Physiology and Chronic Health Evaluation II; SOFA: sequential organ failure assessment.

Data are presented as mean (standard deviation), unless otherwise specified.

^a^Variables are compared across the clusters by the one way analysis of variance, Kruskal–Wallis test, and χ^2^ test as indicated.

### Outcomes

This dataset also leveraged an electronic medical record to continuously recorded data for outcome analysis when patients visited our outpatient department after the index hospitalization. The outcomes of interest were all-cause mortality and being free of dialysis upon 90 days after hospital discharge. All patients were followed up since the initialization of RRT until death, 90 days after hospital discharge or 180 days after the first dialysis, whichever came first. We considered death after weaning off dialysis as mortality, rather than being free of dialysis, because biological kidney recovery without survival is not patient-centred [40].

### Statistical analysis

Continuous variables were presented as mean ± standard deviation, whereas categorical variables were presented as numbers and percentages, unless otherwise stated. To examine the heterogeneity of the patients, we applied consensus clustering based on 23 continuous parameters, namely age, Charlson Comorbidity Index (CCI), baseline eGFR, and other clinical variables upon initializing RRT (all the variables in Table 1B). Clustering was performed using the Gower distance metric and partitioning around medoid algorithm. The optimal cluster size was determined using consensus matrix heat maps, within-cluster consensus scores and delta area plots of the consensus cumulative distribution function [18].

Characteristics were compared among the groups using one-way analysis of variance, the Kruskal–Wallis test, the two-sample t-test, the Mann–Whitney U-test and the chi-square test, as indicated. We plotted Kaplan–Meier curves and built multivariate Cox regression models to determine associations between patient mortality and cluster memberships and clinical variables. Since death is a competing event for kidney recovery, we performed competing risk analysis. Cluster membership and clinical variables were analysed using multivariate Fine–Gray sub-distribution hazard models for being free of dialysis. The cumulative incident function (CIF) was used to estimate the probability of being free of dialysis while treating death as a competing risk [41]. To indicate the implications of mortality against lactate levels, a generalized additive mode incorporating subject-specific (longitudinal) random effects and adjusted for clinical factors was plotted. Furthermore, stepwise logistic regression modelling was applied to establish clinical models for identifying the cluster memberships. All tests were two-tailed with significance defined by *P* values of less than 0.05. Statistical analyses were performed using R 4.1.1 (The R Foundation for Statistical Computing, Vienna, Austria) with the ConsensusClusterPlus package (version 1.56.0) and Stata version 12 (StataCorp LLC, College Station, Texas, USA).

## Results

### Characteristics of the patients

**Figure 1.**
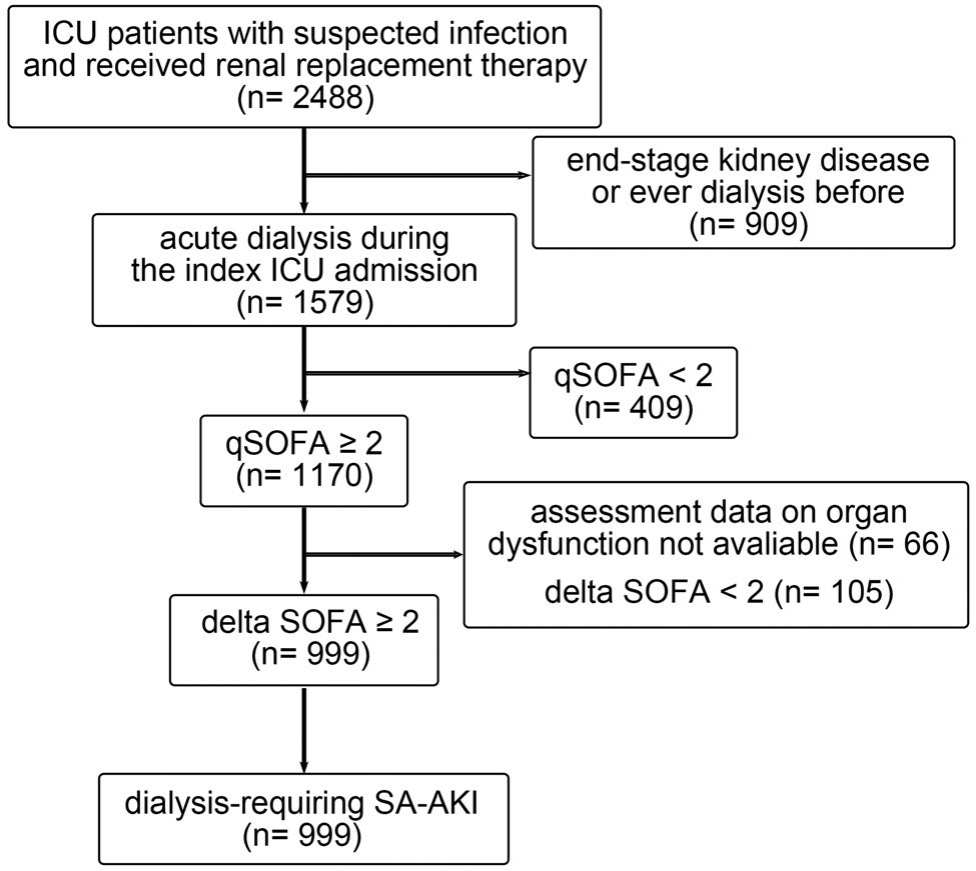
Flow diagram of study population. A total of 2488 critically ill patients who received renal replacement therapy between January 1, 2009 and December 31, 2018 were screened. In total, 999 patients who were diagnosed to have sepsis defined by the Sepsis-3 criteria and dialysis-requiring SA-AKI were enrolled in this study. ICU: intensive care unit; SA-AKI: sepsis-associated acute kidney injury; qSOFA: quick SOFA score; SOFA: sequential organ failure assessment score.

Among the 2488 patients who received RRT and suspected infection during the study period, 909 were excluded because they had ever received acute or maintenance dialysis before the index ICU admission. Of the remaining 1579 patients, 999 who had a quick SOFA score of 2 or higher and had an acute increase in SOFA score of 2 or higher were enrolled in the study (Figure 1). Demographic characteristics were listed in Table 1A. Their mean age was 63.87 ± 16.51 years and 714 (71.47%) patients were male. The modality of the first RRT was continuous renal replacement therapy (CRRT) in 59.86%, sustained low efficiency dialysis in 21.22% and intermittent haemodialysis in 18.92% of the patients. Table 1B shows the clinical data upon initializing RRT. The median IE was 7.7 (interquartile range [IQR] 0–19.25), the median Acute Physiology and Chronic Health Evaluation (APACH) II score was 21 (IQR 17–25), and the median SOFA score was 13 (IQR 10–15).

### Dialysis-requiring SA-AKI sub-phenotypes

Consensus clustering, with cluster size ranging from 2 to 6, was performed to agnostically identify distinct subpopulations of patients (Supplemental Figure S1A–E). Clustering when the cluster size was more than 3 generated one or two clusters with a relative lower mean consensus value, indicating less stability of the cluster membership (Supplemental Figure S1F). The changes in the area under the consensus cumulative distribution function curve did not conspicuously increase when the cluster size was more than 3 (Supplemental Figure S2). Accordingly, we identified three clusters that fairly represented the clinical parameters upon initializing RRT. Cluster 1 comprised 352 (35.24%) patients, and cluster 2 consisted of 396 (39.64%) patients, whereas cluster 3 had 251 (25.13%) patients. The mean consensus value was 0.77 for cluster 1, 0.70 for cluster 2 and 0.85 for cluster 3.

**Figure 2.**
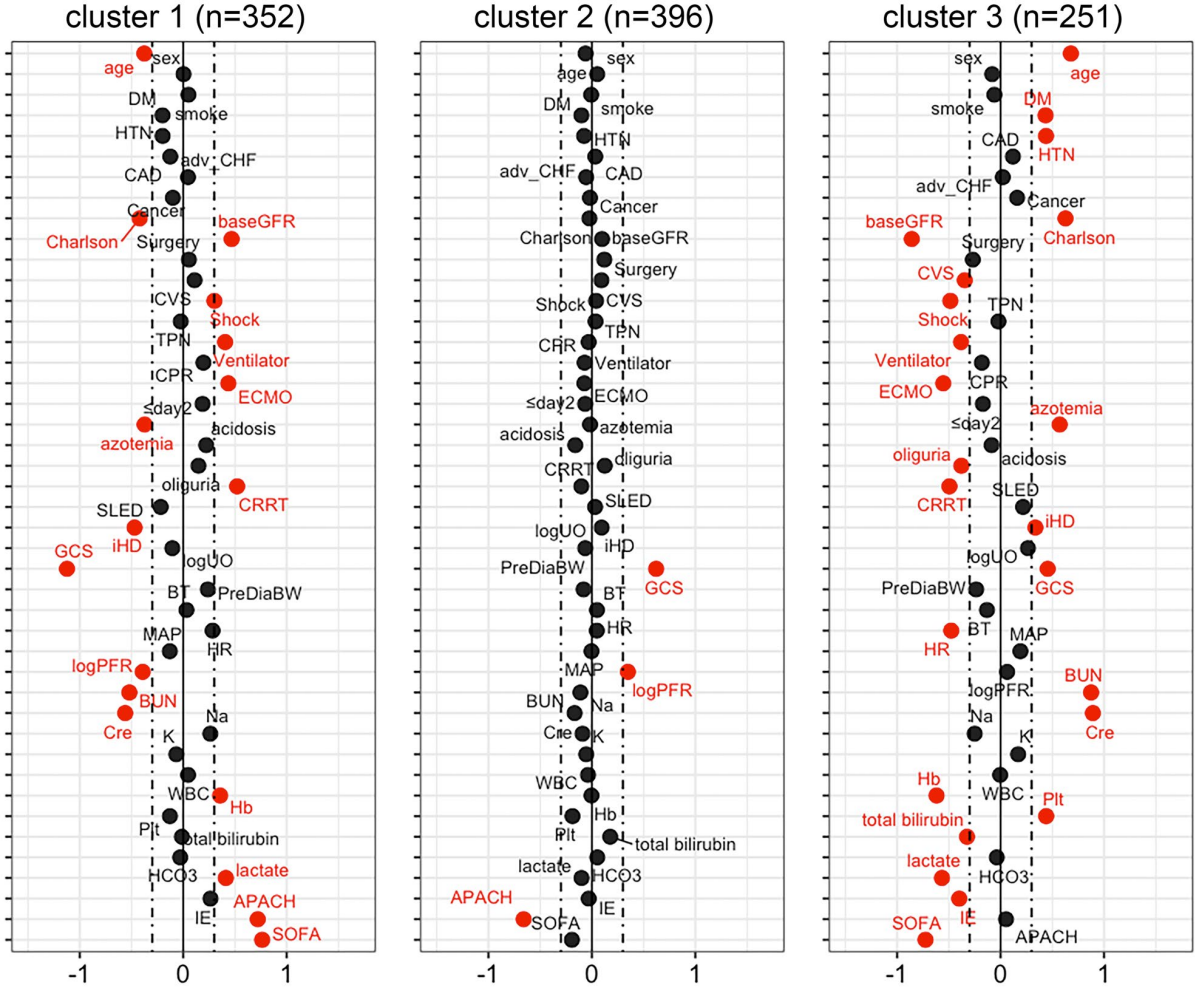
Baseline characteristics across the three phenotypic clusters of patients. The Manhattan plot of the standardized differences in the baseline clinical characteristics across the three clusters revealed by the 23 parameters upon initializing renal replacement therapy. The dashed vertical lines represent the absolute standardized difference of 0.3. Key features (having an absolute standardised difference of ≥0.3) for each cluster are marked in red. DM: diabetes mellitus; HTN: hypertension; CAD: coronary artery disease; adv_CHF: advanced heart failure: defined by New York Heart Association functional class III or IV; Charlson: Charlson Comorbidity Index; baseGFR: baseline estimated glomerular filtration rate; CVS: cardiovascular surgery; TPN: total parenteral nutrition; CPR: cardiopulmonary resuscitation; ECMO: extracorporeal membrane oxygenation; ≤day2: first RRT within 2 days of hospital admission; CRRT: continuous renal replacement therapy; SLED: sustained low efficiency dialysis; iHD: intermittent haemodialysis; logUO: logarithm of urine volume; GCS: Glasgow coma scale; PreDiaBW: body weight before first dialysis; BT: body temperature; HR: heart rate; MAP: mean arterial pressure; logPFR: logarithm of the PaO2 to fraction of inspired oxygen ratio; BUN: blood urea nitrogen; Cre: serum creatinine; Na: sodium; K: potassium; WBC: white blood cells; Hb: haemoglobin; Plt: platelet count; HCO3: bicarbonate; IE: inotropic equivalent; APACH: Acute Physiology and Chronic Health Evaluation II score; SOFA: sequential organ failure assessment score.

The distribution of most baseline characteristics was significantly different across the three clusters, indicating that sub-phenotypes existed in the cohort (Table 1, Supplemental Figure S3 and S4). Figure 2 shows the standardized difference in the baseline characteristics according to each cluster. Key features of the clusters were depicted by having an absolute standardized difference of ≥0.3. Cluster 1 included individuals with favourable underlying conditions, that is, younger age, lower CCI and higher baseline eGFR. Nevertheless, acute clinical status upon initializing RRT was worst in cluster 1, as observed by their highest likelihood of having shock, lowest Glasgow Coma Scale (GCS) and PaO_2_ to fraction of inspired oxygen ratio and higher serum lactate, APACH II and SOFA scores. A higher proportion of them received mechanical ventilation, extracorporeal membrane oxygenation and CRRT as their first RRT. In contrast, cluster 3 comprised individuals with older age, had a higher burden of comorbidities, and lower baseline eGFR. However, the severity of acute illness seems to be lower, suggested by their lower serum lactate, IE and SOFA scores upon initializing RRT. Compared with other clusters, patients in cluster 3 were less likely to receive cardiovascular surgery, but were more likely to initiate RRT due to azotaemia with symptoms and received intermittent haemodialysis as their first RRT.

**Figure 3.**
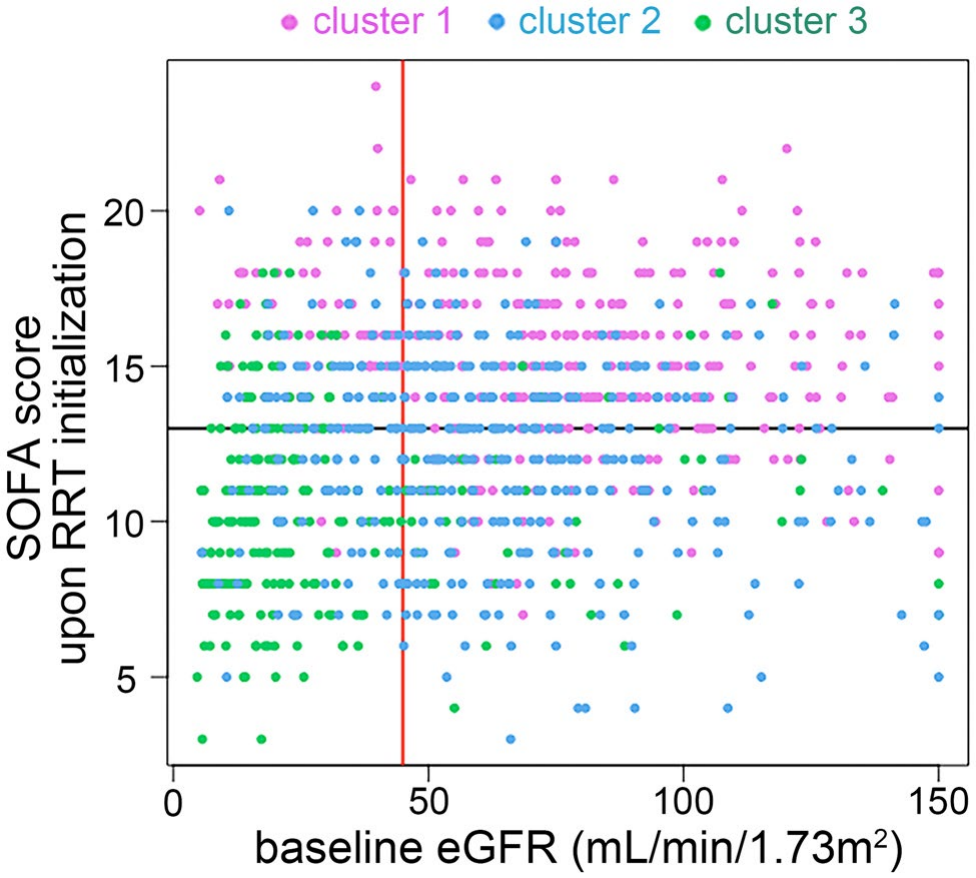
Associations of baseline eGFR and SOFA score upon RRT initialization with the three clusters of patients. The scatter plot illustrates each patient’s baseline eGFR and SOFA score upon RRT initialization, coloured by the three clusters. The red vertical line represents eGFR of 45 mL/min/1.73m^2^; the black horizontal line represents SOFA score of 13. eGFR: baseline estimated glomerular filtration rate; SOFA: sequential organ failure assessment; RRT: renal replacement therapy.

Figure 3 illustrates each patient’s baseline eGFR and SOFA score upon initializing RRT. Most patients in cluster 1 (80.4%) and cluster 2 (70.45%) had baseline eGFR of 45 ml/min/1.73m^2^ or higher, while 79.28% patients in cluster 3 had baseline eGFR lower than 45 ml/min/1.73m^2^. There were 84.38%, 51.26%, and 25.5% patients in cluster 1, 2, and 3 had a SOFA score ≥13, respectively.

### Associations between sub-phenotypes and clinical outcomes

**Figure 4.**
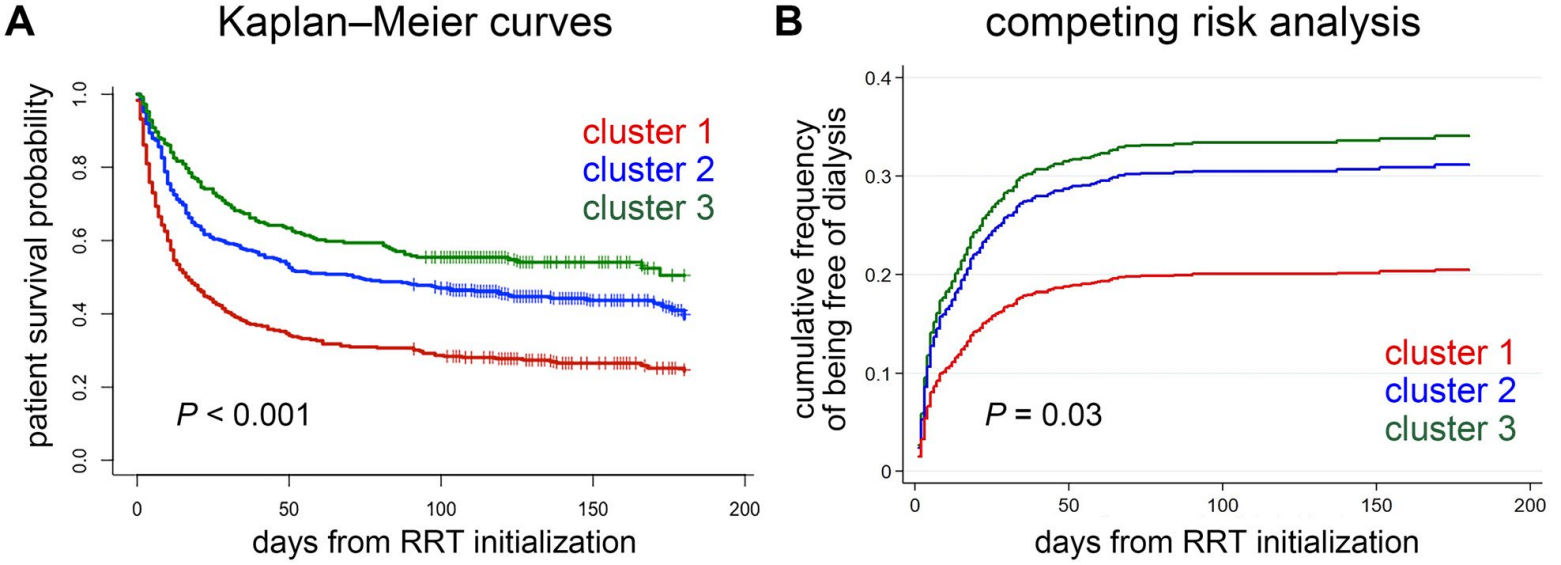
Clinical outcomes across the three clusters of patients. (A) Kaplan–Meier curves depict the survival probability of patients by the three clusters. (B) Cumulative incidence of being free of dialysis stratified by the three clusters with death as the competing risk. RRT: renal replacement therapy.

All enrolled patients were followed up for a median of 48 (IQR, 9.5–128.5) days. All-cause mortality occurred in 600 (60.06%) patients. Moreover, 112 (11.21%) survivors were dialysis dependent, whereas 287 (28.73%) survivors were free of dialysis. Ninety days after hospital discharge, patients in cluster 1 had the highest mortality rate (73.86% vs. 56.57% [cluster 2] vs. 46.22% [cluster 3]; *p* < 0.001). Kaplan–Meier curves showed that survival differences across the three clusters were highly significant (log rank *p* < 0.001) (Figure 4(A)). In unadjusted analysis, cluster 1 (hazard ratio [HR], 2.3; 95% CI, 1.85–2.86) and 2 (HR, 1.36; 95% CI, 1.08–1.7) had elevated mortality risks than cluster 3. After adjusting for age, sex, baseline eGFR and CCI, Cox hazard analysis showed that patients in clusters 1 (adjusted HR, 3.05; 95% CI, 2.35–3.97) and 2 (adjusted HR, 1.65; 95% CI, 1.29–2.11) were associated with an increased risk of death compared with patients in cluster 3.

The crude rate of being free of dialysis 90 days after hospital discharge was different among clusters (25.85% [cluster 1] vs. 33.59% [cluster 2] vs. 25.1% [cluster 3]; *p* = 0.02). The CIF plot demonstrated that the phenotypic cluster was associated with different probabilities of being free of dialysis (Gray’s test *p* = 0.03) (Figure 4(B)). After adjusting for age, sex, baseline eGFR and CCI in the Fine–Gray sub-distribution hazard model, patients in clusters 1 (adjusted sub-distribution hazard ratio [sHR], 0.55; 95% CI, 0.38–0.8), but not in cluster 2 (adjusted sHR 0.9; 95% CI, 0.64–1.25), were less likely to become free of dialysis than those in cluster 3.

**Figure 5.**
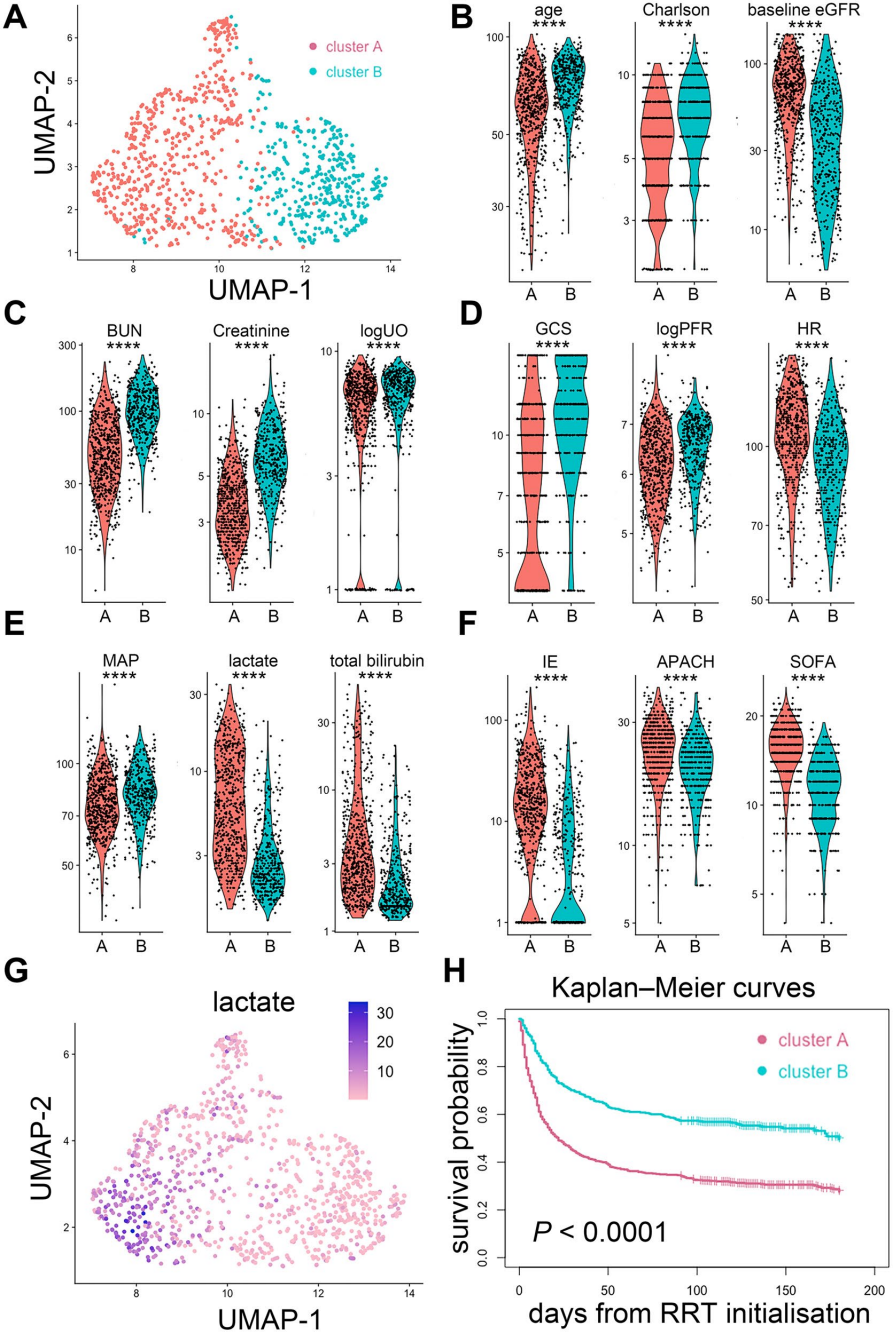
Supplementary unsupervised clustering of patients with dialysis-requiring sepsis-associated acute kidney injury. (A) This clustering approach was performed by principal component analysis of the 23 clinical parameters using the k-nearest neighbour graph structure embedding and Louvain community algorithm. Two clusters (591 patients in cluster A, 408 patients in cluster B) were identified and visualized by the Uniform Manifold Approximation and Projection (UMAP) dimensional reduction. (B-F) Violin plots showed the different features between the two clusters. ****p < 0.0001. (G) Feature plot projected on the UMAP depicts the serum lactate levels upon initializing renal replacement therapy. (H) Kaplan–Meier curves of patient survival stratified by the two clusters. Charlson: Charlson Comorbidity Index; eGFR: estimated glomerular filtration rate; BUN: blood urea nitrogen; logUO: logarithm of urine volume; GCS: Glasgow Coma Scale; BT: body temperature; MAP: mean arterial pressure; logPFR: logarithm of the PaO2 to fraction of inspired oxygen ratio; IE: inotropic equivalent; APACH: Acute Physiology and Chronic Health Evaluation II score; SOFA: sequential organ failure assessment score; RRT: renal replacement therapy.

We performed another supplementary clustering by principal component analysis of the same 23 parameters using the k-nearest neighbour graph structure and Louvain algorithm [28,42]. This approach identified two clusters and revealed a similar observation: a cluster featured by younger age, lower CCI, higher baseline eGFR but with greater severity of acute illness was associated with an increased risk of death; meanwhile, another cluster featured by older age, higher CCI and lower baseline eGFR, but with less severity of acute illness, was associated with better survival (Figure 5).

### Identification of high-risk sub-phenotype

For clinical application, we grouped cluster 2 and 3 together and determined whether a set of clinical variables listed in Table 1 could identify cluster 1, which has a highest risk for mortality and becoming dialysis-dependent. Using stepwise logistic regression, a model composited of 11 variables was developed to accurately identify high-risk sub-phenotype 1, with a C-static 0.99 (Table 2, Supplemental Figure S5). At the cut-off point with optimal Youden index, this model achieved a sensitivity of 95.74%, specificity of 95.83%, positive predictive value of 97.64%, and negative predictive value of 92.59% in the main study cohort.

**Table 2.** Clinical model for identifying high-risk sub-phenotype 1.

Variable	β Coefficient	Odds ratio (95% CI)	*p* Value
Intercept	–23.608		
Charlson	–0.854	0.43 (0.33–0.54)	<0.001
Baseline eGFR, mL/min/1.73m^2^	0.057	1.06 (1.04–1.08)	<0.001
BUN, mg/dL	–0.024	0.98 (0.97–0.99)	<0.001
Cre, mg/dL	–0.312	0.73 (0.57–0.94)	0.02
GCS	–1.318	0.27 (0.20–0.36)	<0.001
BT, degree Celsius	0.661	1.94 (1.36–2.75)	<0.001
HR, beats per minute	0.063	1.07 (1.04–1.09)	<0.001
logPFR	–2.276	0.10 (0.05–0.22)	<0.001
Hb, g/dL	0.526	1.69 (1.38–2.07)	<0.001
APACH II score	0.337	1.40 (1.26–1.56)	<0.001
SOFA score	0.271	1.31 (1.14–1.50)	<0.001
C-static = 0.993			
*R*^2^ = 0.9131			

Charlson: Charlson Comorbidity Index; eGFR: estimated glomerular filtration rate; BUN: blood urea nitrogen; Cre: serum creatinine; GCS: Glasgow Coma Scale; BT: body temperature; HR: heart rate; logPFR: logarithm of the PaO2 to fraction of inspired oxygen ratio; Hb: haemoglobin; APACH II: Acute Physiology and Chronic Health Evaluation II; SOFA: sequential organ failure assessment.

Formula for sub-phenotype 1 = odds/(1 + odds), in which odds = exp (–23.608 – 0.854 × Charlson + 0.057 × baseline eGFR – 0.024 × BUN – 0.312 × Cre – 1.318 × GCS + 0.661 × BT + 0.063 × HR – 2.276 × logPFR + 0.526 × Hb + 0.337 × APACH II + 0.271 × SOFA).

### Pre-dialysis hyperlactatemia ≥3.3 mmol/L predicts poor outcomes

Through the cluster analysis, we identified a sub-phenotype (cluster 1) with poor clinical outcomes and was characterized by a notably higher serum lactate level upon initializing RRT (Figure 2). This association was also evident in the supplementary clustering analyses (Figure 5(G)). Thus, we examined the association of serum lactate level with clinical outcomes. By applying the generalized additive mode with adjustment for age, sex, baseline eGFR, CCI and mean arterial pressure upon initializing RRT, the estimated probability of death augmented when the serum lactate level was equal to or more than 3.3 mmol/L (Supplemental Figure S6A). Multivariable Cox proportional hazards analysis revealed that serum lactate levels of ≥3.3 mmol/L upon initializing RRT independently predict all-cause mortality (adjusted HR, 1.34; 95% CI, 1.09–1.65) (Supplemental Table S1). After controlling for mortality as a competing risk, patients who had hyperlactatemia of ≥3.3 mmol/L upon initializing RRT were less likely to become free of dialysis (adjusted sHR, 0.69; 95% CI, 0.51–0.95) (Supplemental Table S2). Supplemental Figure S6B and C demonstrate that patients with pre-dialysis hyperlactatemia of ≥3.3 mmol/L had poor survival and lower probability of being free of dialysis.

### External cohort

**Figure 6.**
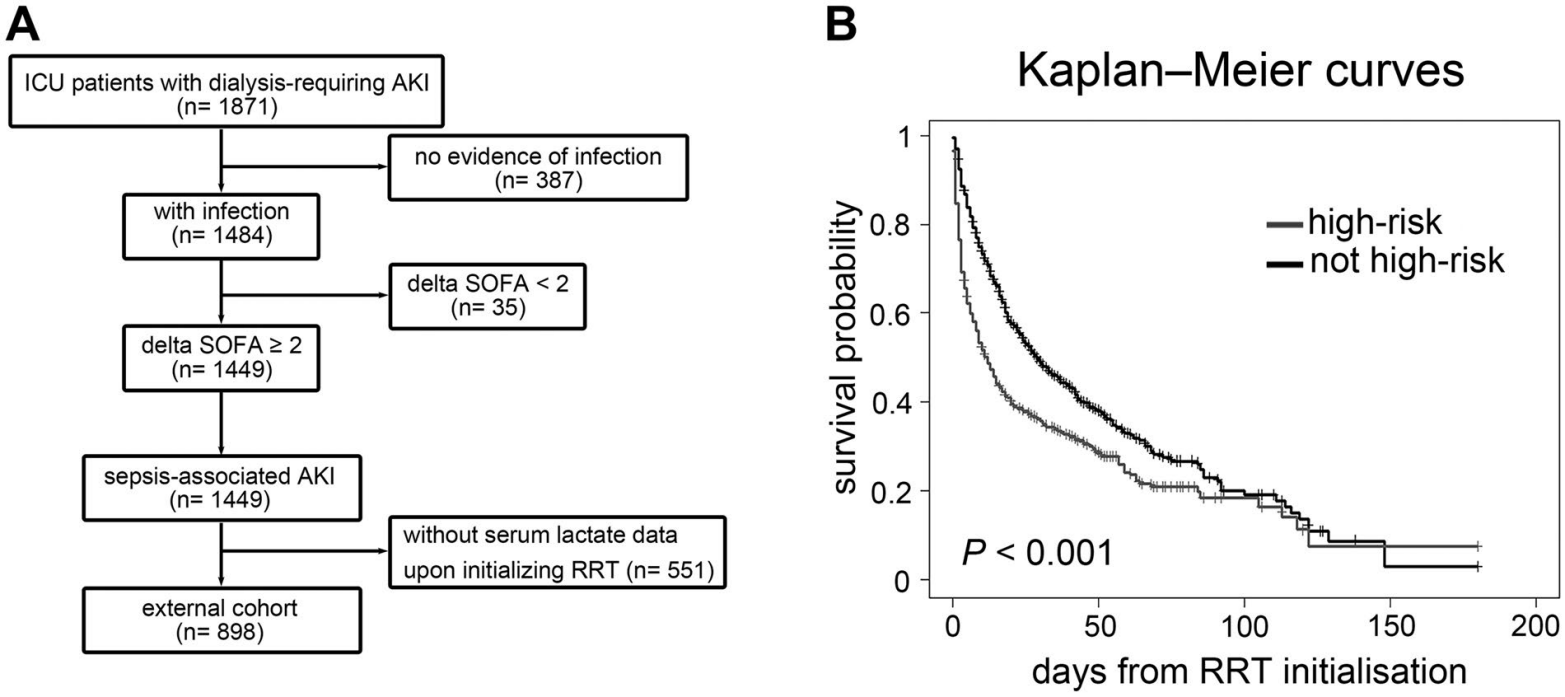
Supplementary unsupervised clustering of patients with dialysis-requiring sepsis-associated acute kidney injury. (A) This clustering approach was performed by principal component analysis of the 23 clinical parameters using the K-nearest neighbour graph structure embedding and Louvain community algorithm. Two clusters (591 patients in cluster A, 408 patients in cluster B) were identified and visualized by the Uniform Manifold Approximation and Projection (UMAP) dimensional reduction. (B-F) Violin plots showed the different features between the two clusters. ****p < 0.0001. (G) Feature plot projected on the UMAP depicts the serum lactate levels upon initializing renal replacement therapy. (H) Kaplan–Meier curves of patient survival stratified by the two clusters. Charlson: Charlson Comorbidity Index; eGFR: estimated glomerular filtration rate; BUN: blood urea nitrogen; logUO: logarithm of urine volume; GCS: Glasgow Coma Scale; BT: body temperature; MAP: mean arterial pressure; logPFR: logarithm of the PaO2 to fraction of inspired oxygen ratio; IE: inotropic equivalent; APACH: Acute Physiology and Chronic Health Evaluation II score; SOFA: sequential organ failure assessment score; RRT: renal replacement therapy.

To extrapolate our findings, we analysed a prospective, observational, multi-centre database of the Nationwide Epidemiology and Prognosis of Dialysis-requiring AKI (NEP-AKI-D) study [5, 7]. The NEP-AKI-D study was conducted in ICUs of the 30 participating hospitals in Taiwan. In our previous report, this dataset included 23% of surgical patients and 77% of medical patients [43]. We extracted a subset of 898 patients who encountered dialysis-requiring SA-AKI and had available serum lactate data upon initializing RRT between 2014 and 2016 (Table 1, Figure 6(A), and Supplemental Methods). After a median follow-up of 17.5 (IQR, 6–39) days, all-cause hospital mortality occurred in 588 (65.48%).

By applying the clinical model derived from the main study cohort (Table 2), 319 (35.53%) patients in the external cohort were classified as high-risk sub-phenotype. The Kaplan–Meier curves showed that high-risk sub-phenotype was associated with lower survival upon hospital discharge (log rank *p* < 0.001)(Figure 6(B)). After adjusting for age, sex, baseline eGFR and CCI, Cox hazard analysis showed that high-risk sub-phenotype assignment was associated with an increased risk of in-hospital mortality (adjusted HR, 1.48; 95% CI, 1.25–1.74).

## Discussion

There is no specific effective therapy for AKI, partly due to its diverse clinical presentations and not well-understood pathophysiology. One personalized approach is the phenotypical- and/or biomarkers-based managements to prevent AKI or to timely treat AKI [24,44,45]. This study presents another approach to inform the planning of managements at the time of RRT initiation, when critical treatment decisions are often being made. We performed consensus clustering in critically ill patients with dialysis-requiring SA-AKI and identified phenotypic clusters. Importantly, the revealed sub-phenotypes showed relevant association with patient mortality and being free of dialysis. Moreover, this data-driven approach led us to discover hyperlactatemia ≥3.3 mmol/L to be an independent outcome predictor.

Recently, clustering algorithms are increasingly used to figure out heterogeneity in AKI based on patients’ demographic features, clinical data, laboratory values and/or biomarkers at ICU admission [24–26]. We focused on patients suffering with the most severe form of AKI, namely dialysis-requiring SA-AKI. Consistent with previous reports, our results highlighted the clinical utility of using unsupervised cluster discovery to explore clinically meaningful sub-phenotypes. Of note, although patients in cluster 1 were younger, had lower CCI and had higher baseline eGFR, they had worse disease severities upon initializing RRT and poor clinical outcomes. Similarly, Chaudhary and colleagues have previously revealed a sub-phenotype in which patients were the youngest, had lowest proportions of hypertension, congestive heart failure, and diabetes, but had most severe acute illness and highest mortality [26]. This suggests that the impact of acute-illness severity outweighs that of baseline general condition in patients with dialysis-requiring SA-AKI.

Compared with prior studies on non-dialysis-requiring SA-AKI [24–26], our study revealed few different findings. First, baseline severity of acute illness (APACH II and SOFA scores, Table 1B) and mortality were much higher in our patients with dialysis-requiring SA-AKI. Even in this extremely vulnerable population, heterogeneity was still present. There was a ‘relatively less risky’ sub-phenotype 3 and a ‘very high-risk’ sub-phenotype 1. Our developed model for identifying high-risk sub-phenotype in dialysis-requiring SA-AKI could be applied in clinical care, such as performing bedside risk calculations by APP- or web-based risk calculator. It may be useful to estimate patient prognosis and help clinical decision-making. Additionally, we demonstrated that patients in sub-phenotype 3 had worse baseline eGFR (Table 1A) but had better outcomes compared with those in sub-phenotype 1. It is possible that encountering dialysis-required SA-AKI in patients with higher baseline kidney function represents a more significant loss of functioning nephrons due to more severe injury, compared to those with poor baseline kidney function. This is compatible with clinical observations that outcomes of severe dialysis-requiring AKI versus underlying advance chronic kidney disease needing dialysis in ICU are very different [46,47]. Furthermore, in our study, sub-phenotype 1 included more patients receiving first RRT within 2 days of hospital admission (Table 1A and Supplemental Figure 3 W) and had worst outcome. Conversely, Wonnacott and colleagues showed that patient with community-acquired AKI had better clinical outcomes than those with hospital-acquired AKI [48]. This discrepancy may be related to different patient profiles between the studies. Besides, there is difficulty in identifying the exact onset of AKI in sepsis [15]. Those who received RRT later than 2 days of hospitalization might have encountered kidney injury earlier in the course of sepsis.

By constellating patients’ comorbidity profiles and acute-illness severities upon initializing RRT, the cluster membership provided a simple prediction of outcomes. Our data suggests that sub-phenotypes in ICU patients with dialysis-requiring SA-AKI may respond differently to ‘usual care’. Interestingly, Bhatraju and colleagues have demonstrated that a certain biomarker-based sub-phenotype of AKI benefited from vasopressin therapy, while others did not [24]. Similarly to research in AKI, differential treatment response to fluid management strategy between sub-phenotypes of acute respiratory distress syndrome has also been reported [49]. This clustering approach can be used as a practical measure for prognostication, help to tailor therapeutic strategies, and facilitate precise patient recruitment in future clinical trials.

Another advantage of unsupervised clustering is its potential to discover previously unrecognized or unconfirmed predictive factors [25]. It has been reported that hyperlactatemia is an independent mortality predictor in patients with SA-AKI in the emergency department [50], postoperative AKI requiring RRT[29] and SA-AKI requiring CRRT [9]. However, other studies have shown that the initial lactate level is not independently associated with death in patients with SA-AKI requiring CRRT [10,51] or post-cardiovascular surgery AKI [32]. Moreover, in the literature, serum lactate has never been reported as an independent predictor of kidney recovery after AKI [6,29,52,53]. In this study, consensus clustering demonstrated that serum lactate levels upon initializing RRT were a disparate feature among clusters (Figure 2). Patients with serum lactate levels of ≥3.3 mmol/L upon initializing RRT had not only a higher risk of mortality but also a higher risk of dialysis dependence, independent of blood pressure, the dose of vasoactive agents and disease severity (Supplemental Table S1 and S2). Whether hyperlactatemia of ≥3.3 mmol/L, rather than the traditional threshold of 2 mmol/L, should be considered an indication of dialysis initiation or surrogate of disease severity for SA-AKI requires further clinical investigations.

This study has limitations. First, we did not include circulating and/or urinary biomarkers in unsupervised clustering. Prior studies have shown that the accuracy of identifying sub-phenotypes with distinct underlying pathophysiology would be improved by incorporating biomarkers of inflammation, endothelial dysfunction, renal tubular injury and cell cycle arrest [24,25,52,54]. Although all enrolled patients in this study had clinically apparent SA-AKI, the exact pathophysiological mechanisms on AKI were not adjudicated. Since biomarker-guided clinical managements are getting popular, we advocate future investigations to characterize these biomarkers across SA-AKI sub-phenotypes. Second, we investigated patients with dialysis-requiring SA-AKI in the same ethnical ICU. Therefore, the findings of the study cannot be generalized to less severe non-dialysis-requiring AKI or other populations with differently distributed features. Third, we used the worst clinical values measured during the 24-h period before RRT initiation. This may have led to selection bias and misclassification bias because biochemical and physiological values always change rapidly in critically ill patients. Our data-driven cluster analysis approach directly relied on the input of the data. Using different inclusive variables at different time points may result in discrete sub-phenotypes. Lastly, the observational nature of this study without a pre-specified protocol of intervention cannot conclude any causal relationship. We are unable to explore whether sub-phenotypes in dialysis-requiring SA-AKI response differently to a specific treatment. Whether sub-phenotypes of patients and/or pre-dialysis hyperlactatemia would permit earlier intervention and the mitigation of mortality is unclear and requires further evaluations.

## Conclusions

Unsupervised consensus clustering in patients with dialysis-requiring SA-AKI revealed sub-phenotypes with clinical relevance and serves a novel outcome predictor. This data-driven approach also led us to discover that serum lactate levels of 3.3 mmol/L or more upon initializing RRT is an independent prognostic factor. A developed model for identifying high-risk sub-phenotype based on clinical variables upon RRT initiation could be applied in clinical care. Future researches of SA-AKI sub-phenotypes are warranted to characterize biomarkers, to investigate treatment responses, and to validate in other populations. This strategy represents further development toward precision medicine.

## Supplementary Material

Supplemental Material

## Data Availability

The data that support the findings of this study are available from the corresponding author (Prof. Vin-Cent Wu, e-mail q91421028@ntu.edu.tw) on reasonable request.
