# Correction

**DOI:** 10.1080/07853890.2023.2288985

**Published:** 2024-01-03

**Article title:** Unsupervised clustering identifies sub-phenotypes and reveals novel outcome predictors in patients with dialysis-requiring sepsis-associated acute kidney injury

**Authors**: Lai, C.F., Liu, J.-H., Li-Jung Tseng, L.-J., Chun-Hao Tsao, C.-H., Chou, N.- K., Lin, S.-H., Chen, Y.-M., & Wu, V.-C.

**Journal:** Annals of Medicine

**Bibliometrics:** Volume 55, Number 01, pages 2197290

**DOI:** https://doi.org/10.1080/07853890.2023.2197290

When this article was first published online, the legend of Figure 6 went wrong as the legend of Figure 5 was wrongly inserted for Figure 6 although the correct legend was supplied by the author during the proof corrections stage.

The legend of Figure 6 has now been corrected as below:

**Figure 6.** NEP-AKI-D cohort. (A) Among the 1871 patients with dialysis-requiring AKI and hospitalized in ICU between 2014 and 2016, 898 patients with dialysis-requiring sepsis-associated AKI were analyzed as the external cohort. (B) By applying the clinical model derived from the main study cohort, 319 patients in the external cohort were categorized as high-risk sub-phenotype. Kaplan–Meier curves showed that patients classified as having a high-risk sub-phenotype had higher in-hospital mortality compared to other patients. NEP-AKI-D: nationwide epidemiology and prognosis of dialysis-requiring acute kidney injury study; AKI, acute kidney injury; ICU, intensive care unit; SOFA, sequential organ failure assessment score; RRT, renal replacement therapy.

