# Supplementary material for: Unsupervised clustering identifies sub-phenotypes and reveals novel outcome predictors in patients with dialysis-requiring sepsis-associated acute kidney injury
Source: Ann Med. 2023 Apr 12;55(1):2197290. doi: 10.1080/07853890.2023.2197290 (PMC10101673; doi:10.1080/07853890.2023.2197290)
Supplement: Supplemental Material [file IANN_A_2197290_SM7106.docx]

**Unsupervised clustering identifies sub-phenotypes and reveals novel outcome predictors in patients with dialysis-requiring sepsis-associated acute kidney injury**

Chun-Fu Lai, MD, PhD; Vin-Cent Wu, MD, PhD; Jung-Hua Liu, PhD; Shuei-Liong Lin, MD, PhD; Yung-Ming Chen, MD

**Supplemental material**

Supplemental Methods ……………………………………………………………… 3

Supplemental Figure S1. Consensus matrix heat maps and cluster-consensus plots… 6

Supplemental Figure S2. Consensus cumulative distribution function plots………… 7

Supplemental Figure S3. Comparisons of baseline demographic characteristics across the three subphenotypes of patients with dialysis-requiring sepsis-associated acute kidney injury ………………………………………………………………………… 8

Supplemental Figure S4. Comparisons of baseline clinical characteristics upon initialising RRT across the three subphenotypes of patients with dialysis-requiring sepsis-associated acute kidney injury………………………………………………… 9

Supplemental Figure S5. Area under the receiver operator characteristics curve for sub-phenotype 1 identification……………………………………………………… 10

Supplemental Figure S6. Associations of serum lactate levels and outcomes in patients with dialysis-requiring sepsis-associated acute kidney injury …………… 11

Supplemental Table S1. Multivairable-adjusted analysis for independent predictors for mortality ………………………………………………………………………… 12

Supplemental Table S2. Analysis for independent factors associated with being free of dialysis using the competing risk approach……………………………………… 13

**Supplemental methods**

**Clinical assessments of patients**

All data in the National Taiwan University Hospital Study Group on Acute Renal Failure (NSARF) database were prospectively collected on a standardized form.[1] We extracted the following demographic data obtained at intensive care unit (ICU) admission: age, sex, smoking history, diabetes mellitus (defined by using oral antidiabetic agents or insulin), hypertension (using anti-hypertensive drugs or systolic and diastolic blood pressure >140/90 mmHg), coronary artery disease (defined by the diagnostic code of ischemic heart disease prior to admission and positive electrocardiographic findings), stroke (ischemic or hemorrhagic), peripheral arterial occlusive disease (defined by clinical or imaging diagnosis), advanced heart failure (defined as New York Heart Association functional class III or IV), chronic obstructive pulmonary disease (with long-term bronchodilators), and liver cirrhosis (defined by image studies with computed tomography or sonography). Cancer was recorded according to diagnostic codes prior to admission. Patients with associated diseases were assessed using the Charlson Comorbidity Index.[2]

The worst physiological and laboratory values during the 24-hour period before the first renal replacement therapy (RRT) were collected. Serum lactate levels upon initializing RRT were missing in 132 (13.21%) of individuals. All other variables were missing for 0.5% or less (urine volume 2 [0.2%], ratio of PaO2 to fraction of inspired oxygen 1 [0.1%], blood urea nitrogen 3 [0.3%], white blood cells count 4 [0.4%], platelets count 5 [0.5%], total bilirubin 4 [0.4%], bicarbonate 3 [0.3%], inotropic equivalent 3 [0.3%]). Missing data was imputed using Multivariate Imputation by Chained Equation (MICE). The severity scores including the Glasgow Coma Scale, the Sequential Organ Failure Assessment score,[3] and the Acute Physiology and Chronic Health Evaluation II score were measured. The doses of inotropic therapies (expressed as inotropic equivalent [μg/kg/min] = dopamine + dobutamine + 100×epinephrine + 100×norepinephrine + 100×isoprotenolol + 15×milrinone),[4] usages of mechanical ventilation, total parenteral nutrition, surgery, and extracorporeal membrane oxygenation were also recorded.

**Dialysis initiation**

The indications for RRT initiation included but not limited to: (i) azotemia, defined as blood urea nitrogen >80 mg/dL or serum creatinine >2 mg/dL, with uremic syndrome, such as confusion, platelet dysfunction, pericarditis, or uremic pneumonitis; (ii) refractory fluid overload, defined by a central venous pressure level >12 mmHg or pulmonary arterial wedge pressure >18 mmHg; (iii) refractory hyperkalemia >5.5 mmol/L (iv) refractory metabolic acidosis with a pH <7.2; (v) oliguria (urine output <400 mL/24h) or anuria refractory to diuretics. The modality of dialysis was documented as either continues renal replacement therapy, sustained low efficiency dialysis, or intermittent hemodialysis according the attending physician and adjusted by the critical care nephrologists based on the disease evolutions.

**The NEP-AKI-D study**

The nationwide epidemiology and prognosis of dialysis-requiring acute kidney injury (NEP-AKI-D) study was launched by the Consortium for Acute Kidney Injury and Renal Disease (CAKs) in Taiwan. This observational study prospectively enrolled critically ill adult patients with dialysis-requiring acute kidney injury (AKI) in ICUs of the 30 participating hospitals. These hospitals distribute through the four geographic regions (north, middle, south, and east) of Taiwan and have a 1:1 ratio of tertiary medical centers to regional hospitals in each geographic region.[5] All adults patients encountering dialysis-requiring AKI, defined by the Kidney Disease: Improving Global Outcomes criteria, in the seasonal sampled months (January, April, July, and October in study period) were enrolled. The exclusion criteria included patients who received chronic dialysis before the index hospitalization, and died within 48 hours after admission to the ICU. The National Research Program for Biopharmaceuticals-Institutional Review Board (NRPB2014050014) and institutional review boards of each hospital approved this study and waived the need for informed consents because all personal information is de-identified. The NEP-AKI-D study has been described previously.[5-8] Many of the participating ICUs are medical, cardiac, and mixed ICUs. In our previous report, this dataset included 23% of surgical patients and 77% of medical patients.[8]

**External cohort and patients’ outcomes**

In the NEP-AKI-D study cohort, there were 1871 critically ill patients encountering dialysis-requiring AKI between January 2014 and December 2016. Among them, we used Sepsis-3 criteria[3] to define sepsis and identified 1449 patients with sepsis-associated AKI. Comparing with other collected clinical variables in the NEP-AKI-D study, serum lactate measurements upon initializing RRT were less available. Five hundred and fifty-one patients were excluded from analyses due to the lack of pre-dialysis serum lactate data. Finally, 898 patients were included as an external replication cohort (Table 1, Figures 6A).

Demographic and clinical characteristics of the 898 patients are listed in Table S2. They were followed since the initialization of RRT until death, hospital discharge, or 180 days after the first dialysis, whichever came first. After a median follow-up of 17.5 (interquartile range 6-39) days, all-cause hospital mortality occurred in 588 (65.48%). There were 84 (9.35%) patients were alive in dialysis dependency, while 226 (25.17%) were alive and wean from dialysis upon hospital discharge.

**Reference to the supplemental methods**

1. Wu VC, Huang TM, Lai CF, et al. Acute-on-chronic kidney injury at hospital discharge is associated with long-term dialysis and mortality. *Kidney Int*. 2011;80(11):1222-1230.

2. Charlson ME, Pompei P, Ales KL, et al. A new method of classifying prognostic comorbidity in longitudinal studies: development and validation. *J Chronic Dis*. 1987;40(5):373-383.

3. Singer M, Deutschman CS, Seymour CW, et al. The Third International Consensus Definitions for Sepsis and Septic Shock (Sepsis-3). *JAMA*. 2016;315(8):801-810.

4. Chen YS, Lin JW, Yu HY, et al. Cardiopulmonary resuscitation with assisted extracorporeal life-support versus conventional cardiopulmonary resuscitation in adults with in-hospital cardiac arrest: an observational study and propensity analysis. *Lancet*. 2008;372(9638):554-561.

5. Shiao CC, Wu PC, Wu VC, et al. Nationwide epidemiology and prognosis of dialysis-requiring acute kidney injury (NEP-AKI-D) study: Design and methods. *Nephrology (Carlton)*. 2016;21(9):758-764.

6. Chen YY, Wu VC, Huang WC, et al. Norepinephrine Administration Is Associated with Higher Mortality in Dialysis Requiring Acute Kidney Injury Patients with Septic Shock. *J Clin Med*. 2018;7(9).

7. Wu VC, Chueh SJ, Chang JT, et al. Acute Kidney Injury and Septic Shock-Defined by Updated Sepsis-3 Criteria in Critically Ill Patients. *J Clin Med*. 2019;8(10).

8. Shiao CC, Chang YH, Yang YF, et al. Association between regional economic status and renal recovery of dialysis-requiring acute kidney injury among critically ill patients. *Sci Rep*. 2020;10(1):14573.

**Supplemental Figure S1. Consensus matrix heat maps and cluster-consensus plots. (A-E)** Consensus matrix heat maps of 999 patients with dialysis-requiring sepsis-associated acute kidney injury. Each pairwise consensus of all participant is color-coded by the blue color gradient scale. The darkest blue color represents perfect consensus when two patients grouped together, while the white color represent perfect consensus when two patients grouped separately. The cluster memberships are marked by colored rectangles between the dendrogram and heat map. Graphics of the consensus matrices are depicted for different cluster size: (A) *k* = 2, (B) *k* = 3, (C) *k* = 4, (D) *k* = 5, (E) *k* = 6. **(F)** Cluster-consensus plots illustrated the mean of all pairwise consensus values between members within each cluster. Clusters are indicated by color marks. The plots are grouped by different cluster size *k* which is marked on the horizontal axis. The mean consensus value on the vertical axis depicts the stability of the clusters.


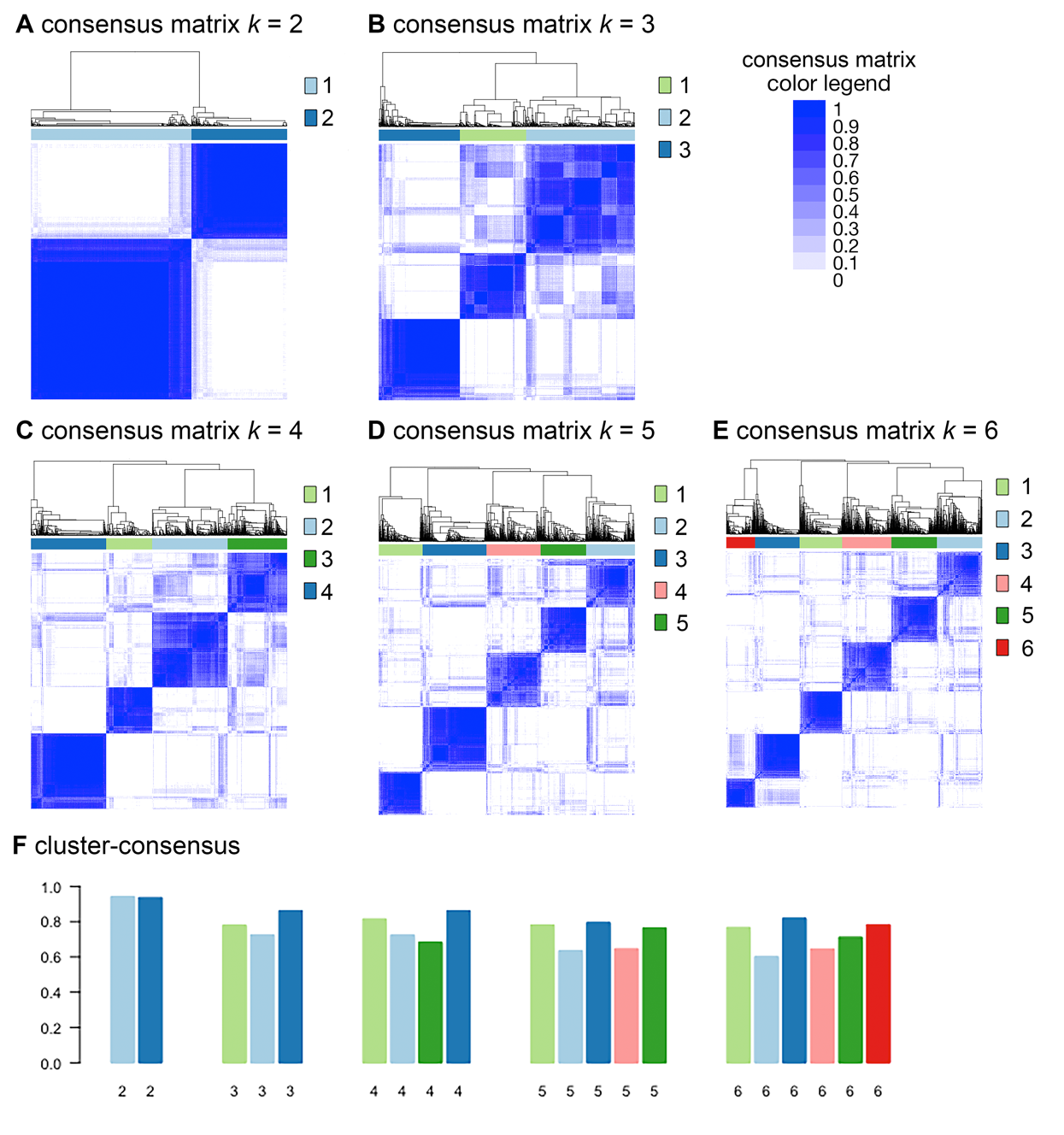


**Supplemental Figure S2. Consensus cumulative distribution function plots.** (A) Consensus cumulative distribution function (CDF) of the consensus matrix estimated by a histogram of 100 bins. Different CDFs for each cluster size *k* are labeled by different colors. (B) Delta area plot shows the changes in the area under the CDF curve comparing cluster size *k* and *k*-1. Of note, the changes of area under CDF curve did not conspicuously increase when the cluster size *k* was more than 3.


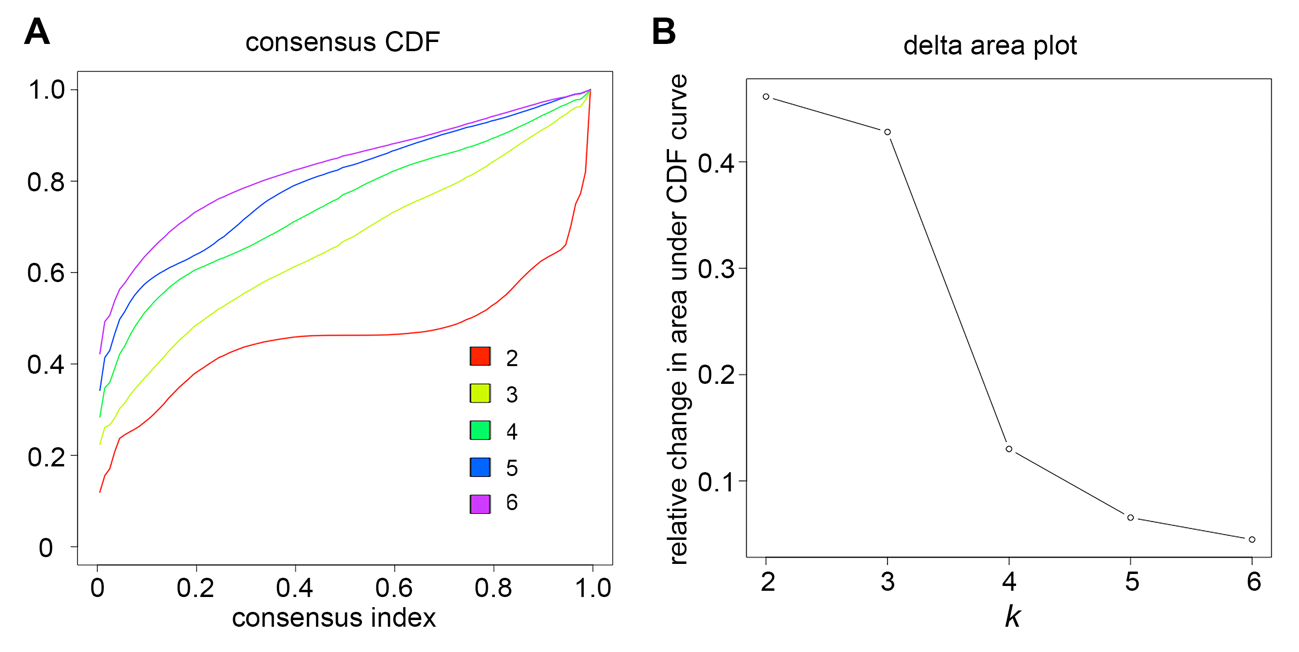


**Supplemental Figure S3. Comparisons of baseline demographic characteristics across the three subphenotypes of patients with dialysis-requiring sepsis-associated acute kidney injury.** Continuous variables are illustrated by violin plots with median (thick lines) and interquartile range (dotted lines). Categorical variables are presented as percentages (cluster 1: red; cluster 2: blue; cluster 3: green). Characteristics were compared among the groups using one-way analysis of variance, the Kruskal–Wallis test, the two-sample t-test, the Mann–Whitney U test, and the chi-square test, as indicated. * *P* <0.05; ** *P* <0.01; *** *P* <0.001; **** *P* <0.0001.


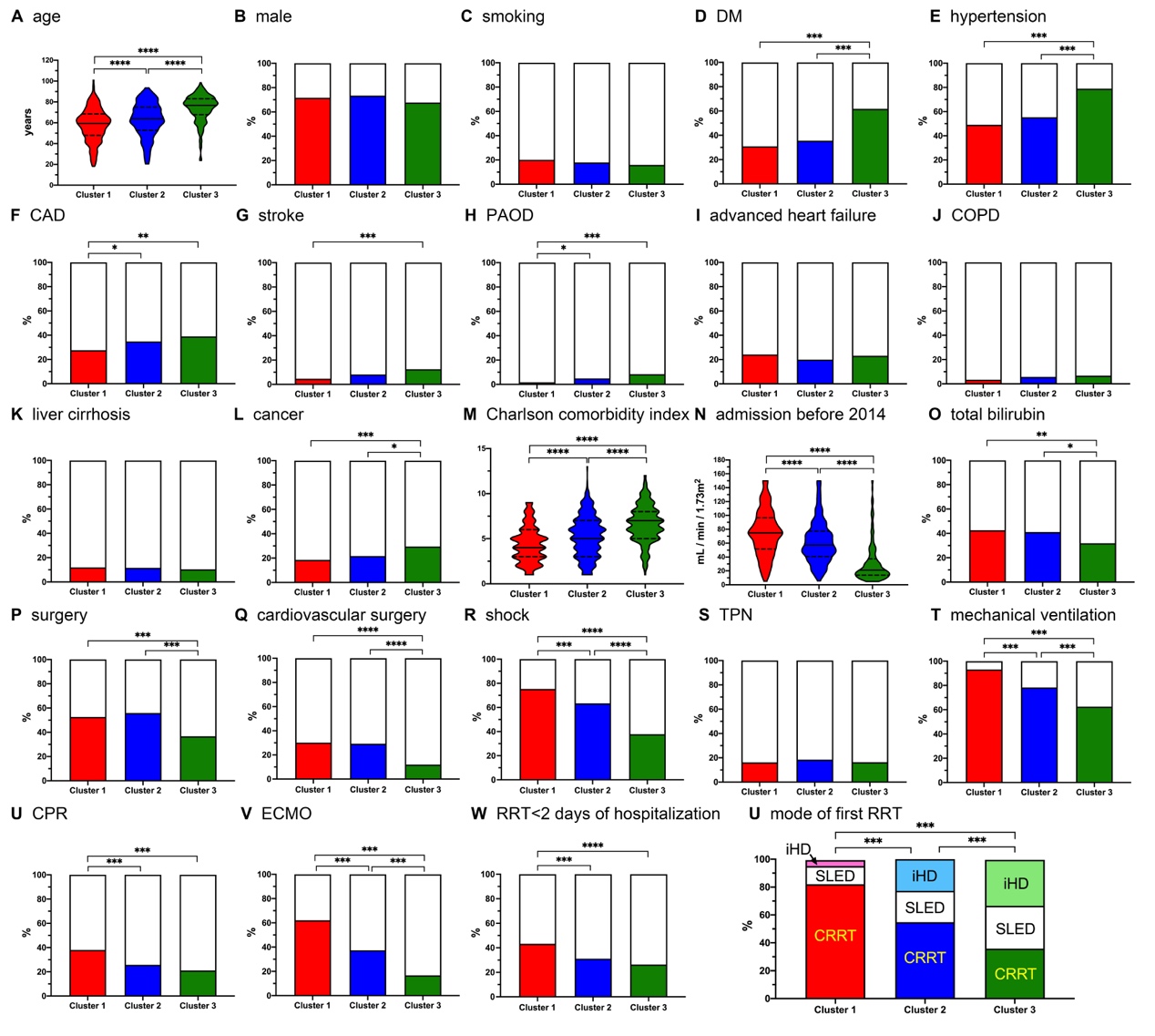


**Supplemental Figure S4. Comparisons of baseline clinical characteristics upon initializing renal replacement therapy across the three subphenotypes of patients with dialysis-requiring sepsis-associated acute kidney injury.** Continuous variables are illustrated by violin plots with median (thick lines) and interquartile range (dotted lines). Categorical variables are presented as percentages (cluster 1: red; cluster 2: blue; cluster 3: green). Characteristics were compared among the groups using one-way analysis of variance, the Kruskal–Wallis test, the two-sample t-test, the Mann–Whitney U test, and the chi-square test, as indicated. * *P* <0.05; ** *P* <0.01; *** *P* <0.001; **** *P* <0.0001.


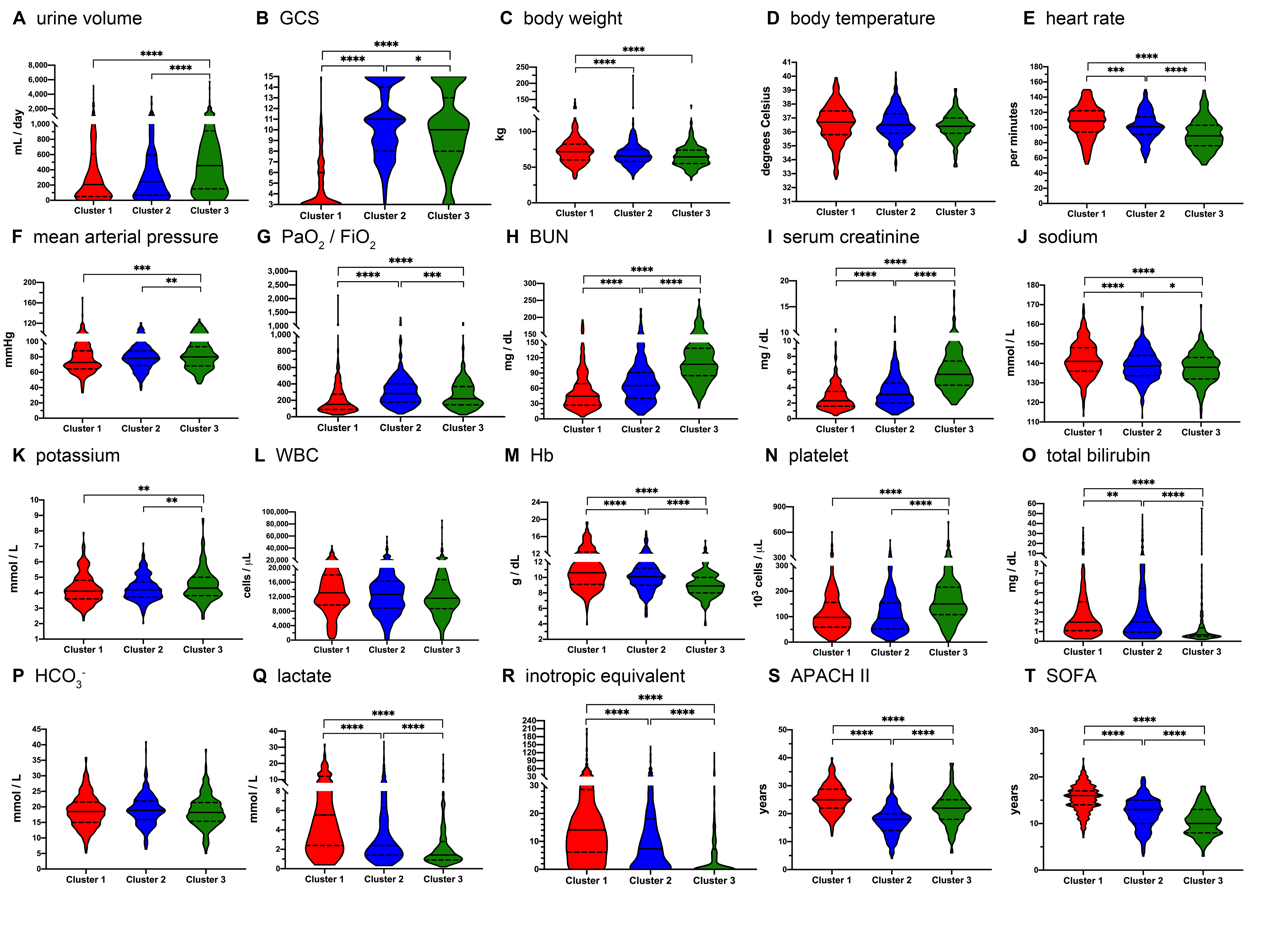


**Supplemental Figure S5. Area under the receiver operator characteristics curve for sub-phenotype 1 identification.**

**
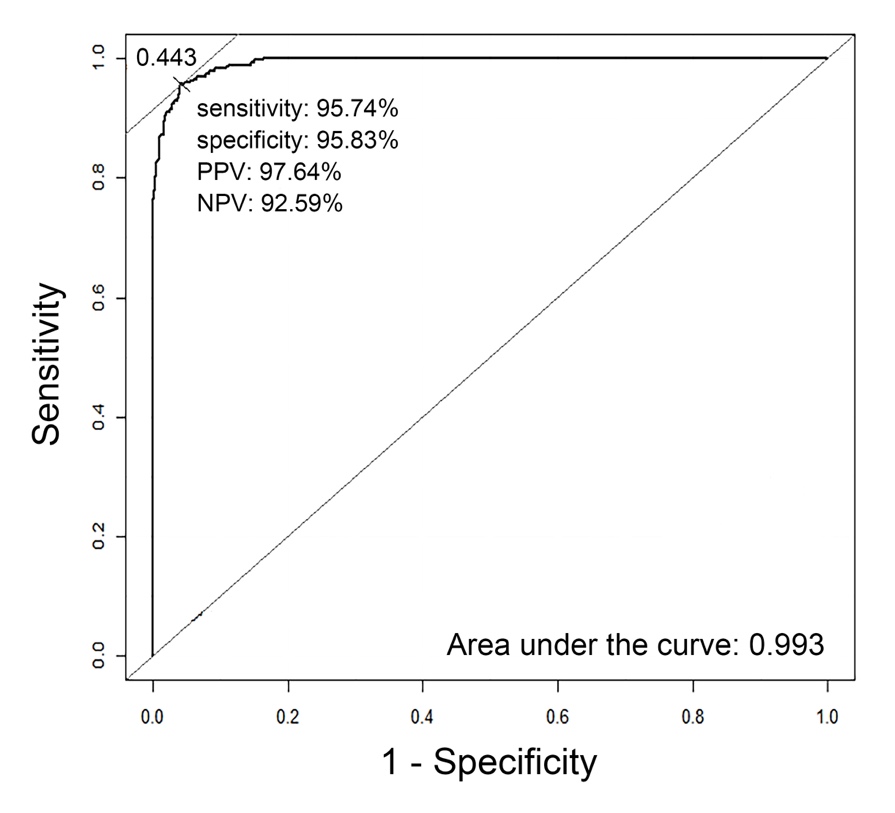
**

Formula for sub-phenotype 1 = odds / (1+odds), in which odds = exp (-23.608 - 0.854×Charlson + 0.057×baseline eGFR - 0.024×BUN - 0.312×Cre - 1.318×GCS + 0.661×BT + 0.063×HR - 2.276×logPFR + 0.526×Hb + 0.337×APACH II + 0.271×SOFA). At the cutoff point 0.443, this model has highest Youden index.

Abbreviations: Charlson, Charlson Comorbidity Index; eGFR, estimated glomerular filtration rate; BUN, blood urea nitrogen; Cre, serum creatinine; GCS, Glasgow Coma Scale; BT, body temperature; HR, heart rate; logPFR, logarithm of the PaO2 to fraction of inspired oxygen ratio; Hb, hemoglobin; APACH II, Acute Physiology and Chronic Health Evaluation II; SOFA, sequential organ failure assessment.

**Supplemental Figure S6. Associations of serum lactate levels and outcomes in patients with dialysis-requiring sepsis-associated acute kidney injury.** (A) The plot was drawn using a generalized additive model with adjustment for age, sex, baseline estimated glomerular filtration rate, Charlson Comorbidity Index and mean arterial pressure upon initializing RRT. The estimated probability of death 90 days after hospital discharge raised gradually accompanying the increase of serum lactate levels upon initializing RRT. (B) The Kaplan–Meier survival analysis for patients with serum lactate level <3.3 mmol/L and ≥3.3 mmol/L upon initializing RRT (log rank test *P* <0.001). (C) Cumulative incidence of being free of dialysis, stratified by the threshold of serum lactate levels of 3.1 mmol/L upon initializing RRT with death as the competing risk (Gray’s test *P* =0.003). (D) The association of hyperlactatemia with mortality was validated in 898 critically ill patients with dialysis-requiring sepsis-associated acute kidney injury from the NEP-AKI-D cohort. Kaplan–Meier survival analysis for patients with serum lactate level <3.3 mmol/L and ≥3.3 mmol/L upon initializing RRT (log rank test *P* <0.001).

Abbreviations: RRT, renal replacement therapy; NEP-AKI-D: nationwide epidemiology and prognosis of dialysis-requiring acute kidney injury study.

**
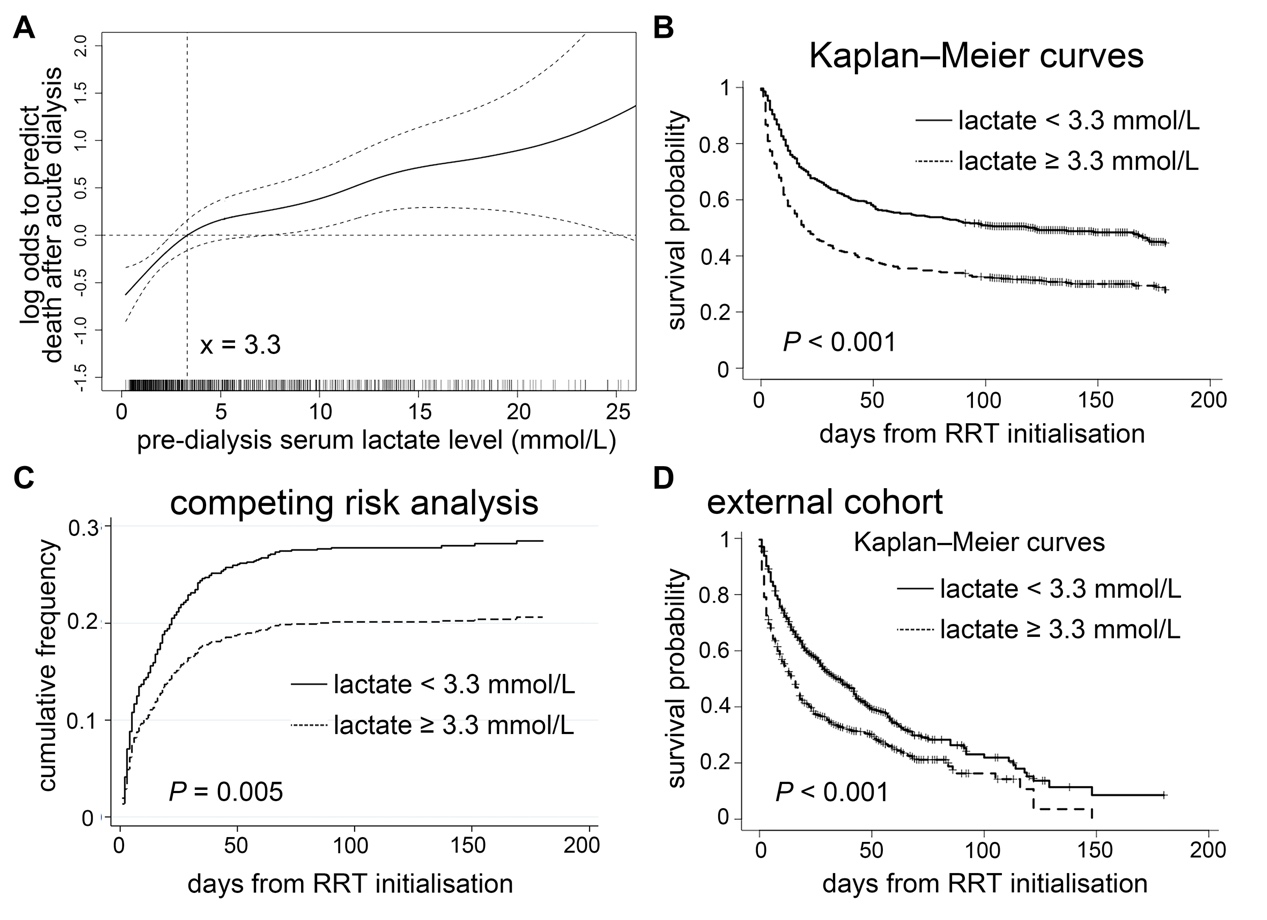
**

**Supplemental Table S1. Multivariable-adjusted analysis for independent predictors for mortality.**

| **Variables** | **adjusted HR^*,^** ^†^ **(95% CI)** | ***P*** |
| --- | --- | --- |
| Cancer | 1.38 (1.07-1.78) | 0.01 |
| Index admission year before 2014 | 1.62 (1.31-1.99) | <0.001 |
| Surgery | 0.73 (0.61-0.88) | <0.001 |
| Dialysis initiation due to symptomatic azotemia | 0.75 (0.61-0.93) | 0.01 |
| Body temperature, degree Celsius | 0.9 (0.83-0.97) | 0.01 |
| Mean arterial pressure, mmHg | 0.99 (0.985-0.997) | 0.003 |
| Blood urea nitrogen, mg/dL | 1.005 (1.002-1.01) | <0.001 |
| Serum creatinine, mg/dL | 0.89 (0.83-0.94) | <0.001 |
| Sodium, mmol/L | 1.01 (1.003-1.03) | 0.01 |
| Total bilirubin, mg/dL | 1.03 (1.01-1.04) | <0.001 |
| Lactate ≥ 3.3 mmol/L | 1.34 (1.09-1.65) | 0.005 |
| APACH II score | 1.03 (1.01-1.06) | 0.01 |
| SOFA score | 1.08 (1.03-1.13) | 0.003 |

^*^Multivariable Cox regression analysis of hazard ratios (HR) for mortality.

^†^Other factors included within both models which are not statistically significant: age, sex, smoking, diabetes, hypertension, coronary artery disease, stroke, peripheral arterial occlusive disease, advanced heart failure, chronic obstructive pulmonary disease, liver cirrhosis, Charlson Comorbidity Index, baseline estimated glomerular filtration rate, total parenteral nutrition, mechanical ventilation, cardiopulmonary resuscitation, extracorporeal membrane oxygenation, source of sepsis, indications of dialysis initiations other than symptomatic azotemia, modality at the first renal replacement therapy, urine volume, Glasgow Coma Scale, body weight, heart rate, ratio of PaO2 to fraction of inspired oxygen, potassium, white blood cells count, hemoglobin, platelets count, bicarbonate, and inotropic equivalent.

Abbreviations: CI: confidence interval.

**Supplemental Table S2. Analysis for independent factors associated with being free of dialysis using the competing risk approach.**

| **Variables** | **adjusted sHR**^*,^ **^†^ (95% CI)** | | ***P*** |
| --- | --- | --- | --- |
| Chronic obstructive pulmonary disease | 1.92 (1.04-3.55) | 0.04 | |
| Cancer | 0.67 (0.46-0.98) | 0.04 | |
| Baseline eGFR, mL/min/1.73m^2^ | 1.01 (1.003-1.012) | 0.001 | |
| Surgery | 1.34 (1.04-1.73) | 0.02 | |
| log(urine volume, mL/day) | 1.12 (1.02-1.22) | 0.01 | |
| Body temperature, degree Celsius | 1.13 (1.01-1.28) | 0.04 | |
| Hemoglobin, g/dL | 1.1 (1.03-1.18) | 0.003 | |
| Total bilirubin, mg/dL | 0.96 (0.93-0.99) | 0.01 | |
| Lactate ≥ 3.3 mmol/L | 0.69 (0.51-0.95) | 0.02 | |

^*^Multivariable Fine-Gray model was used, higher sub-distribution hazard ratio (sHR) implies higher probability of kidney recovery from dialysis, taking mortality as a competing risk.

^†^Other factors included within both models which are not statistically significant: age, sex, smoking, diabetes, hypertension, coronary artery disease, stroke, peripheral arterial occlusive disease, advanced heart failure, liver cirrhosis, Charlson Comorbidity Index, index admission year before 2014, total parenteral nutrition, mechanical ventilation, cardiopulmonary resuscitation, extracorporeal membrane oxygenation, source of sepsis, indications of dialysis initiations, modality at the first renal replacement therapy, Glasgow Coma Scale, body weight, heart rate, mean arterial pressure, ratio of PaO2 to fraction of inspired oxygen, blood urea nitrogen, serum creatinine, sodium, potassium, white blood cells count, platelets count, bicarbonate, inotropic equivalent, Acute Physiology and Chronic Health Evaluation score, sequential organ failure assessment score.

Abbreviations: CI: confidence interval; ns: not significant; eGFR, estimated glomerular filtration rate.
